# The Evaluation of Cytotoxic Properties from CCB-2 Sugar Complexes Against TNBC and Non-TNBC Cells

**DOI:** 10.31557/APJCP.2021.22.1.151

**Published:** 2021-01

**Authors:** Riris Istighfari Jenie, Rohmad Yudi Utomo, Ratna Asmah Susidarti, Dhania Novitasari, Mitsunori Kirihata, Edy Meiyanto

**Affiliations:** 1 *Laboratory of Macromolecular Engineering, Department of Pharmaceutical Chemistry, Faculty of Pharmacy, Universitas Gadjah Mada, Sekip Utara II, 55281 Yogyakarta, Indonesia. *; 2 *Cancer Chemoprevention Research Center, Faculty of Pharmacy, Universitas Gadjah Mada, Sekip Utara II, 55281 Yogyakarta, Indonesia. *; 3 *Laboratory of Medicinal Chemistry, Department of Pharmaceutical Chemistry, Faculty of Pharmacy, Universitas Gadjah Mada, Sekip Utara II, 55281 Yogyakarta, Indonesia. *; 4 *Research Center of Boron Neutron Capture Therapy, Osaka Prefecture University, 1-1 Gakuen-cho, Nakaku, Sakai, Osaka, Japan. *

**Keywords:** CCB-2, sugar complexes, cytotoxic, breast cancer

## Abstract

**Objective:**

The progress from Boron Neutron Capture Therapy (BNCT) development urged us to explore new targeted and selective boron carriers. Firstly, we reported the successful synthesis of CCB-2 which exerts a cytotoxic effect against triple negative breast cancer (TNBC) cells. We introduced the new modification of CCB-2 with sugar and alcohol sugars to enhance its solubility in hoping to increase cellular uptake.

**Methods:**

CCB-2 fructose complex (CCB-2-F), CCB-2 sorbitol complex (CCB-2-Sor), and CCB-2 xylitol complex (CCB-2-Xy) were obtained with small size within nano-specific particle. All the compounds were then determined for their cytotoxic activities through MTT assay.

**Results:**

All compounds were performed cytotoxic activities against TNBC 4T1 and HER-2 positive MCF-7/HER2 cells with good selectivity when tested in immortalized fibroblast cells.

**Conclusion:**

Overall, we provided a new modification of CCB-2 through complexation with sugars. Still, further evaluations are needed to develop more efficient CCB-2 as the new candidate of anticancer agent, notably in breast cancer.

## Introduction

**Figure 1:**
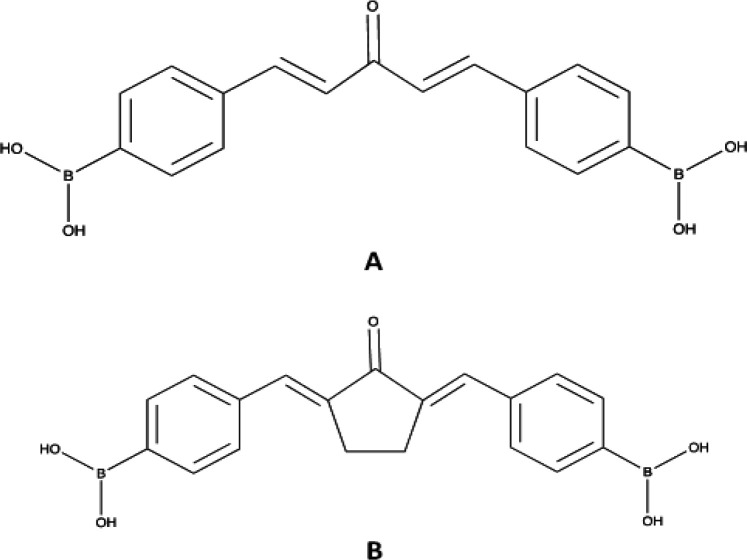
Chemical Structure of CCB-2 (A) and Pentagamaboronon-0 (B)

Previously, we have successfully synthesized CCB-2 (3-oxopenta-1,4-diene-1,5-diylbis-4-phenylboronic acid) (Figure 1A), a new boron bearing compound (BCC) named which is preferred as the novel compound with good cytotoxic effect against cancer cells, notably in TNBC (Meiyanto et al., 2020). The cytotoxic activity from CCB-2 demonstrates much better compared with 200-fold superior against another BCC Pentagamaboronon-0 (PGB-0) (Figure 1B) (Kusumastuti et al., 2019). Therefore, CCB-2 itself can be considered as novel candidate for anticancer drug. Conversely, the very low water solubility and cellular uptake remain as challenge in the application of most curcumin analogues including CCB-2 (Cunico et al., 2017; Susidarti et al., 2019). Hence, the modification of synthesis method and developing of more soluble derivatives of CCB-2 urged to be introduced.

Another possibility on improving CCB-2 solubility is using of monosaccharide or sugar alcohol, which is successfully applied for Boronophenylalanine (L-BPA) and PGB-0 (Shull et al., 2000; Susidarti et al., 2019). Recently, we explored the application of fructose, sorbitol, and xylitol to be added with CCB-2 through complexation in order to enhance the solubility of curcumin analogues, in hoping to increase the bioavailability of the drug. The cytotoxic evaluation of the complex of CCB-2 compound was also determined by testing against two different model of breast cancer: TNBC and HER-2 positive cancer cells, as well as tested in non-cancerous cells to determine the selectivity of these compounds. Through this study, we hope to elucidate the possibility of using CCB-2 and its complexes as boron carrier for BNCT.

## Materials and Methods

*Materials and Tools*

All of the materials and reagents mentioned in the experiments were classified as analytical reagent grade. CCB-2 compound is retrieved from Cancer Chemoprevention Research Center, Universitas Gadjah Mada, Indonesia as reported in Meiyanto et al., (2020), while fructose, sorbitol, and xylitol were purchased from Merck (Germany). 

*Preparation of CCB-2 Complexes*

Boron compounds in complex with fructose, sorbitol, and xylitol were formulated followed by the previous method with slight modification (Susidarti et al., 2019). The 0.184 mmol of fructose, sorbitol, or xylitol was diluted in 23 mL of water. Briefly CCB-2 (0.023 mmol) was added. After stirring for 1 h, NaOH 10% was added dropwise until reached pH 9 and subsequently stirred again for 1 h. The pH was adjusted to 7.5 using Dowex® 50WX4 resin followed by lyophilization. The particle size of all CCB-2 complexes were measured using particle size analyzer.

*Cell Culture*

We used two model of breast cancer cell lines and one fibroblast cell culture throughout this study. The 4T1 breast cancer cell and 3T3 fibroblast cell were kindly given by Prof. Masashi Kawaichi (NAIST, Japan), while MCF-7/HER2 breast cancer cell line was kindly gifted from Prof. Yoshio Inouye (Toho University, Japan) through Prof. Masashi Kawaichi (NAIST, Japan). All of cells were maintained in high glucose-DMEM (Gibco, New York, USA) mixed with 10% (v/v) fetal bovine serum (FBS) (Gibco, New York, USA), 4-(2hydroxyethyl)-1-piperazineethanesulfonic acid (HEPES), sodium bicarbonate, 1.5% Peniciliin-Streptomycin (Gibco, New York, USA). Cells were grown at 37 °C incubator supported with 5% CO_2_.

*Cytotoxicity Assay*

We performed MTT assay to assess the cytotoxic activity of CCB-2 complexes with slight modification (Mosmann, 1983). MCF-7/HER2 (5 × 10^3^ cells/well) and 4T1 cells (2 × 10^3^ cells/well), and also 3T3 cells cells (1 × 10^4^ cells/well) were seeded separately in 96-well microplates and cultured for 24 h. CCB-2 fructose (CCB-2-F), CCB-2 sorbitol (CCB-2-Sor), and CCB-2 xylitol (CCB-2-Xy) were given to each well and incubated for 24 h at several concentrations. After treatment, cells were stained by 0.5 mg/mL of MTT (Biovision, California, USA) and incubated for next 4 h. The enzymatic reaction were stopped with sodium dodecyl sulfate (SDS) (with 0.01 N HCl) and stored in the dark condition overnight. The absorbance was quantified by microplate reader plate at 595 nm (Corona SH-1000, Corona Electric Co. Ltd., Ibaraki-ken, Japan). The absorbance was formulated to percent of cell viability. Linear regression between concentration and percent of cell viability were used to calculate the half inhibitory concentration or IC_50_ value of each compound. Later, the selectivity index was calculated by using equation as mentioned in Prayong et al. (2008).

**Table 1:** Particle Size and Isoelectric Point Profile of CCB-2-F, CCB-2-Sor, and CCB-2-Xy

Compound	Particle Size (nm)	Isoelectric Point
CCB-2-F	149.7	0.594
CCB-2-Sor	106	0.295
CCb-2-Xy	146.1	0.429

**Figure 2:**
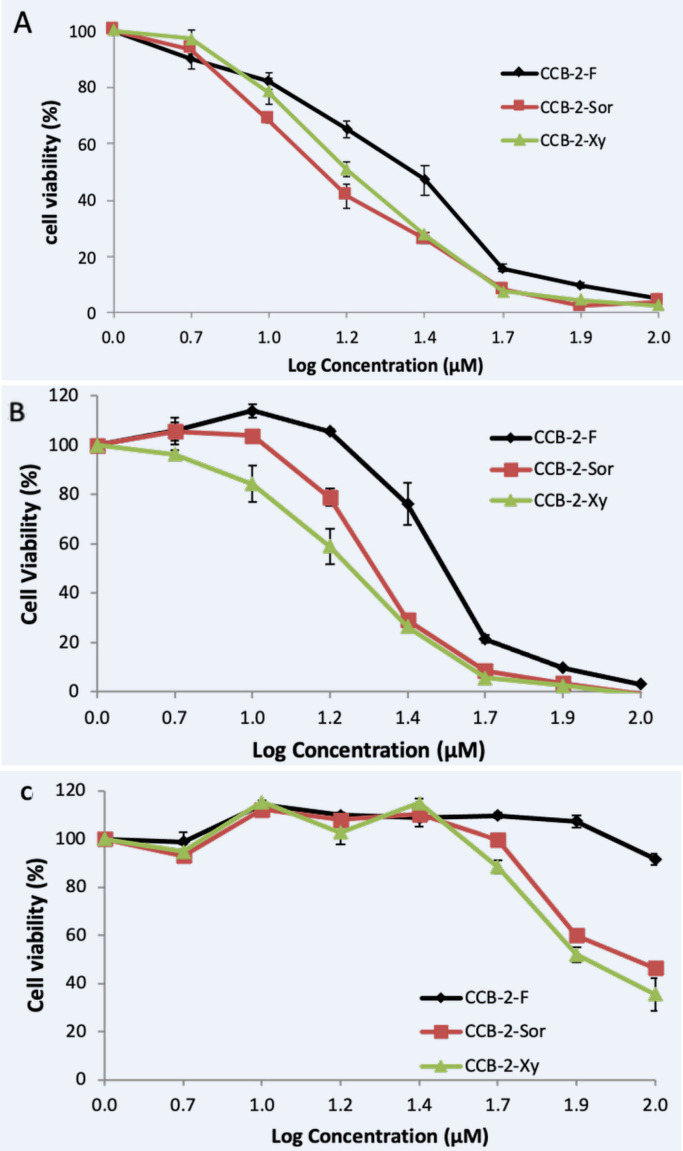
Cytotoxicity of CCB-2-F, CCB-2-Sor, and CCB-2-Xy on Several Cell Lines, Including 4T1 (A), MCF-7/HER-2 (B), and 3T3 (C) Cells. Cells were treated by all tested compounds for 24 h as described in the method section. The graph represents mean ± SE from three independent experiments

**Table 2:** Selectivity Index of CCB-2 Complexes against Several Cancer Cells

	Selectivity Index (SI)	
Compound	4T1 cells	MCF-7/HER2 cells
CCB-2-F	>5	>3
CCB-2-Sor	6	4
CCB-2-Xy	5	4

## Results

*The Complexation of CCB-2 using Fructose, Sorbitol, and Xylitol*

Strategy by using ketonic monosaccharide fructose and sugar alcohol (i.e : sorbitol and xylitol) are broadly used to augment the solubility and bioavailability of BPA in water (Kumar et al., 2013; Shull et al., 2000; Watanabe et al., 2016). The complexation is possible to be formed because the presence of boronic acid in CCB-2 structure. After conducting several formulas, the ratio of CCB-2 to fructose, sorbitol, and xylitol as 1:8 were found to be more appropriate compared to other ratio (data not shown). All the complexation compounds are possessed smaller particle within nano-scale with the size of 149.7; 106.0; and 146.1 nm, subsequently (Table 1). Since the CCB-2 alone performed strong cytotoxic activities when tested in TNBC cell model, we then evaluated the effect of all complexed compounds against several types of cancer cells as pointed out earlier.

*Cytotoxicity Study of CCB-2 Complexes in Breast Cancer Cell Lines*

We investigate the potency of CCB-2 complexes for their anticancer activity to consider the ability of our compounds as boron carrier. Our preliminary results revealed CCB-2 complexes performed cytotoxic activities on 4T1 and MCF-7/HER2, but exhibited no cytotoxic effects against fibroblast-like 3T3 cells (Figure 2). Among all tested compound, CCB-2-Sor performed most cytotoxicity potency compared with CCB-2-F and CCB-2-Xy complexes when treated in 4T1 cells with the respective IC_50_ values of 16, 22, and 18 µM. Surprisingly, CCB-2-Xy exerts most potent when observed on MCF-7/HER2 with the IC_50_ values of 19 µM, and 24 µM for CCB-2-Sor, and 37 µM for CCB-2-F. We also calculate the selectivity index (SI) score to determine the selectivity of CCB-2 complexes in cancer cells, and all complexes compounds have SI value more than 2 (Table 2). Since in several publications stated that the anticancer candidate is said to have good selectivity when scored SI value greater than 2 (Prayong et al., 2008; Machana et al., 2011), indicated that CCB-2-F, CCB-2-Sor, and CCB-2-Xy perform good selectivity against cancer cells.

## Discussion

The new modification of CCB-2 using sugar and polyol lead to more soluble CCB-2 in water. The complexes using sugars provide more eligible and efficient BNCT application due to the using of simple materials for the production, compared to liposomal and salt modification (Barth et al., 2018). The possible mechanism of complexation with sugar is through binding with boron atom in boronic acid, allowing the boron bearing compound (BCC) to be more soluble in water. The modification is also allowed to form nanoparticle size, which hopefully can enhance the solubility and penetrate more particle into tumor tissue, as well as candidate for new boron carrier (Pulagam et al., 2019).

To our knowledge, boron carrying pharmaceutical (BCP) exhibit low toxicity on normal cells in the BNCT implementation (Barth et al., 2012), our synthesized compounds were examined for their cytotoxicity effect using two different subtype of breast cancer cell line: MCF-7/HER2 (HER2 overexpression) and 4T1 (TNBC), meanwhile for the selectivity toward normal cells, we used 3T3 cells. Breast cancer cases remain majorly occurred in women, with 30% of all cases detected as HER-2 positive. HER-2 itself is part of class of tyrosine kinase receptor that critical for cancer cells proliferation, progression, and moreover metastasis (Dai et al., 2016; Labidi et al., 2016). In contrary, TNBC subtype also promote high mortality on cancer patients due to the lack of hormonal and targeted chemotherapy to treat this subtype, and lead to poor prognosis (Al-Mahmood et al., 2018; Tariq and Rana, 2013). The results showed that our compounds exerted selective target in breast cancer cell lines, while they showed no toxic effect against normal cell models. In addition, cellular cytotoxicity is also possibly mediated through the increase of uptake in the cancer cells (Hattori et al., 2014; Miyahara et al., 1993). Hence, the cellular uptake assay for micro-distribution of drug in cells need to be carried out in next studies. Taken together, CCB-2 is successfully modified into CCB-2-F, CCB-2-Sor, and CCB-2-Xy complexes which bearing nano-sized particles. All complexes performed good cytotoxic agents against breast cancer cells with good selectivity. Hence, CCB-2 and its complexes exhibit their broad possibility to be developed either as BNCT and also as anticancer agent for breast cancer therapy.

## References

[B1] Al-Mahmood S, Sapiezynski J, Garbuzenko OB, Minko T (2018). Metastatic and triple-negative breast cancer: Challenges and treatment options. Drug Deliv Transl Res.

[B2] Barth RF, Vicente MGH, Harling OK (2012). Current status of boron neutron capture therapy of high grade gliomas and recurrent head and neck cancer. Rad Oncol.

[B3] Barth RF, Mi P, Yang W (2018). Boron delivery agents for neutron capture therapy of cancer. Cancer Commun (Lond).

[B4] Cunico LP, Acosta MC, Turner C (2017). Experimental measurements and modeling of curcumin solubility in CO2-expanded ethanol. J Supercrit Fluids.

[B5] Dai X, Xiang L, Li T, Bai Z (2016). Cancer hallmarks, biomarkers and breast cancer molecular subtypes. J Cancer.

[B6] Hattori Y, Kusaka S, Mukumoto M (2014). Synthesis and in vitro evaluation of thiododecaborated α, α- cycloalkylamino acids for the treatment of malignant brain tumors by boron neutron capture therapy. Amino Acids.

[B7] Kumar SK, Sushma M, Raju PY (2013). Dissolution enhancement of poorly soluble drugs by using complexation technique-a review. J Pharm Sci Res.

[B8] Kusumastuti R, Utomo RY, Khumaira A (2019). Pentagamaboronon-0 increased cytotoxicity of and inhibited metastasis induction by doxorubicin in breast cancer cells. J Appl Pharm Sci.

[B9] Labidi S, Mejri N, Lagha A (2016). Targeted therapies in HER2-overexpressing metastatic breast cancer. Breast Care (Basel).

[B10] Machana S, Weerapreeyakul N, Barusrux S (2011). Cytotoxic and apoptotic effects of six herbal plants against the human hepatocarcinoma (HepG2) cell line. Chin Med.

[B11] Meiyanto E, Susidarti RA, Jenie RI (2020). Synthesis of new boron containing compound (CCB-2) based on curcumin structure and its cytotoxic effect against cancer cells. J Appl Pharm Sci.

[B12] Miyahara T, Ueda K, Akaboshi M (1993). Hyperthermic enhancement of cytotoxicity and increased uptake of cis-diamminedichloroplatinum(II) in cultured human esophageal cancer cells. Jpn J Canc Res.

[B13] Mosmann T (1983). Rapid colorimetric assay for cellular growth and survival: application to proliferation and cytotoxicity assays. J Immunol Methods.

[B14] Prayong P, Barusrux S, Weerapreeyakul N (2008). Cytotoxic activity screening of some indigenous Thai plants. Fitoterapia.

[B15] Pulagam KR, Gona KB, Gómez-Vallejo V (2019). Gold nanoparticles as boron carriers for boron neutron capture therapy: Synthesis, radiolabelling and in vivo evaluation. Molecules.

[B16] Shull BK, Spielvogel DE, Head G (2000). Studies on the structure of the complex of the boron neutron capture therapy drug, L-p-boronophenylalanine, with fructose and related carbohydrates: Chemical and 13C NMR evidence for the β-D-fructofuranose 2,3,6-(p-phenylalanylorthoboronate) structure. J Pharm Sci.

[B17] Susidarti RA, Utomo RY, Qodria L (2019). Preparation of pentagamaboronon-0 and its fructose and sorbitol complexes as boron carrier for boron neutron capture therapy (BNCT) application. Res Pharm Sci.

[B18] Tariq K, Rana F (2013). TNBC vs Non-TNBC: A five-year retrospective review of differences in mean age family history smoking history and stage at diagnosis at an Inner city university program. World J Clin Oncol.

[B19] Watanabe T, Hattori Y, Ohta Y (2016). Comparison of the pharmacokinetics between L-BPA and L-FBPA using the same administration dose and protocol: A validation study for the theranostic approach using [18 F]-L-FBPA positron emission tomography in boron neutron capture therapy. BMC Cancer.

